# Exosome isolation from distinct biofluids using precipitation and column-based approaches

**DOI:** 10.1371/journal.pone.0198820

**Published:** 2018-06-11

**Authors:** Tânia Soares Martins, José Catita, Ilka Martins Rosa, Odete A. B. da Cruz e Silva, Ana Gabriela Henriques

**Affiliations:** 1 Neuroscience and Signalling Group, Department of Medical Sciences, Institute of Biomedicine (iBiMED), University of Aveiro, Aveiro, Portugal; 2 CEBIMED—Faculty of Health Sciences; University Fernando Pessoa, Porto, Portugal; 3 Paralab SA, Gondomar, Portugal; 4 The Discovery CTR, University of Aveiro Campus, Aveiro, Portugal; University of Cincinnati College of Medicine, UNITED STATES

## Abstract

The potential of exosomes as biomarker resources for diagnostics, prognostics and even for therapeutics is an area of intense research. Despite the various approaches available, there is no consensus with respect to the best methodology for isolating exosomes and to provide substantial yields with reliable quality. Differential centrifugation is the most commonly used method but it is time-consuming and requires large sample volumes, thus alternative methods are urgently needed. In this study two precipitation-based methods and one column-based approach were compared for exosome isolation from distinct biofluids (serum, plasma and cerebrospinal fluid). Exosome characterization included morphological analyses, determination of particle concentration, stability and exosome preparations’ purity, using different complementary approaches such as Nanoparticle Tracking Analysis, Electrophoretic Light Scattering, Transmission Electron Microscopy, EXOCET colorimetric assay, protein quantification methods and western blotting. The three commercial kits tested successfully isolated exosomes from the biofluids under study, although ExoS showed the best performance in terms of exosome yield and purity. Data shows that methods other than differential centrifugation can be applied to quickly and efficiently isolate exosomes from reduced biofluid volumes. The possibility to use small volumes is fundamental in the context of translational and clinical research, thus the results here presented contribute significantly in this respect.

## Introduction

Exosomes are small spherical, lipid bilayer membrane extracellular vesicles (EVs), ranging from 30 to 150 nm in diameter. These EVs are secreted upon the fusion of multivesicular bodies (MVBs),with the plasma membrane [1,2]. Exosomes can be released by various cell types and found in distinct biofluids such as blood [3,4], saliva [5], cerebrospinal fluid (CSF) [6], urine [7], breast milk [5], human semen [8] and synovial fluid [9]. These nanovesicles represent mechanisms of intercellular communication with several lines of evidence supporting their involvement in biological and pathogenic processes such as in cancer [10–12] and, more recently, in neurodegenerative diseases [13–15]. Exosomes carry a vast proteomic content, but include other components like lipids [16,17] and also a genetic cargo [18]. The fact that these nanovesicles can carry specific protein signatures linked to disease pathogenesis has increased research interest in exosomes as potential resources for biomarker discovery, useful in diagnostics and therapeutics. Exosomes can cross the blood brain barrier enhancing their potential in biomarker discovery for neurodegenerative disease but also as drug delivery vehicles, especially in the blood and CSF [19,20]. However, exosome isolation from biofluids is still a challenge. Differential centrifugation is the most commonly used technique and considered the gold standard for general exosome isolation [21]. This technique results in relatively pure exosomes but recovery rates are low, the procedure is time-consuming, requires appropriate equipment and apparently lacks reproducibility between laboratories [22]. Another relevant aspect in exosome isolation from biological fluids is sample viscosity (e.g. plasma) that requires longer ultracentrifugation times and higher speeds which can compromise exosome integrity [23,24]. Besides ultracentrifugation, many other isolation methods exist, such as filtration, size exclusion chromatography, immunoaffinity isolation, microfluidic devices and polymer-based precipitation techniques [24,25]. Column-based protocols that employ size exclusion chromatography and result in several vesicle fractions, produce exosomes with greater purity, but are time-consuming and provide diluted exosome preparations [26,27]. The use of polymer-based precipitation reagents is increasing as these approaches can provide an easy isolation method that does not require specialized equipment, and render in good exosome yields. In this case, an aspect to consider is if these exosomal preparations, and in particular the reagent itself or the polymer-based reagents co-precipitate contaminants that can interfere with downstream analyses [26]. This technique is based on mixing the biofluid with the polymer containing solution; facilitating exosome precipitation at lower centrifugation speeds rather than ultracentrifugation, thus providing a rapid alternative method [25].

The work here presented compares different isolation methods from distinct biofluids, contributing to the characterization of exosome isolation methods. It compares the performance of two exosome-precipitation based kits and one column-based method to isolate exosomes from different biofluids, namely serum, plasma and CSF. Exosomal preparations were characterized in terms of size, morphology, exosome yield and stability. All methods isolated exosomes, although in terms of yield and purity differences could be observed. The characterization of efficient alternative methods for exosome isolation from biofluids can impact research in the areas of biomarker discovery and translational clinical research.

## Materials and methods

### Biofluids preparation

Biofluids were obtained from the pcb-Cohort biobank collection and ongoing international collaborations [28,29]. Blood samples were collected in tubes with gel separator, with or without EDTA, and centrifuged at 1800 g or 2000 g during 15 min for plasma and serum, respectively. CSF was obtained by lumbar puncture and centrifuged at 1000g for 5 min. All samples were aliquoted and stored at -80°C. This work is part of a project approved by the Ethics Committee for Health of the Central Regional Administration of Coimbra (CES da ARS Centro, protocol No. 012804–04.04.2012) and by the National Committee for Data Protection (CNPD N° 369/2012).

### Exosome isolation from biofluids

**Fig 1:**
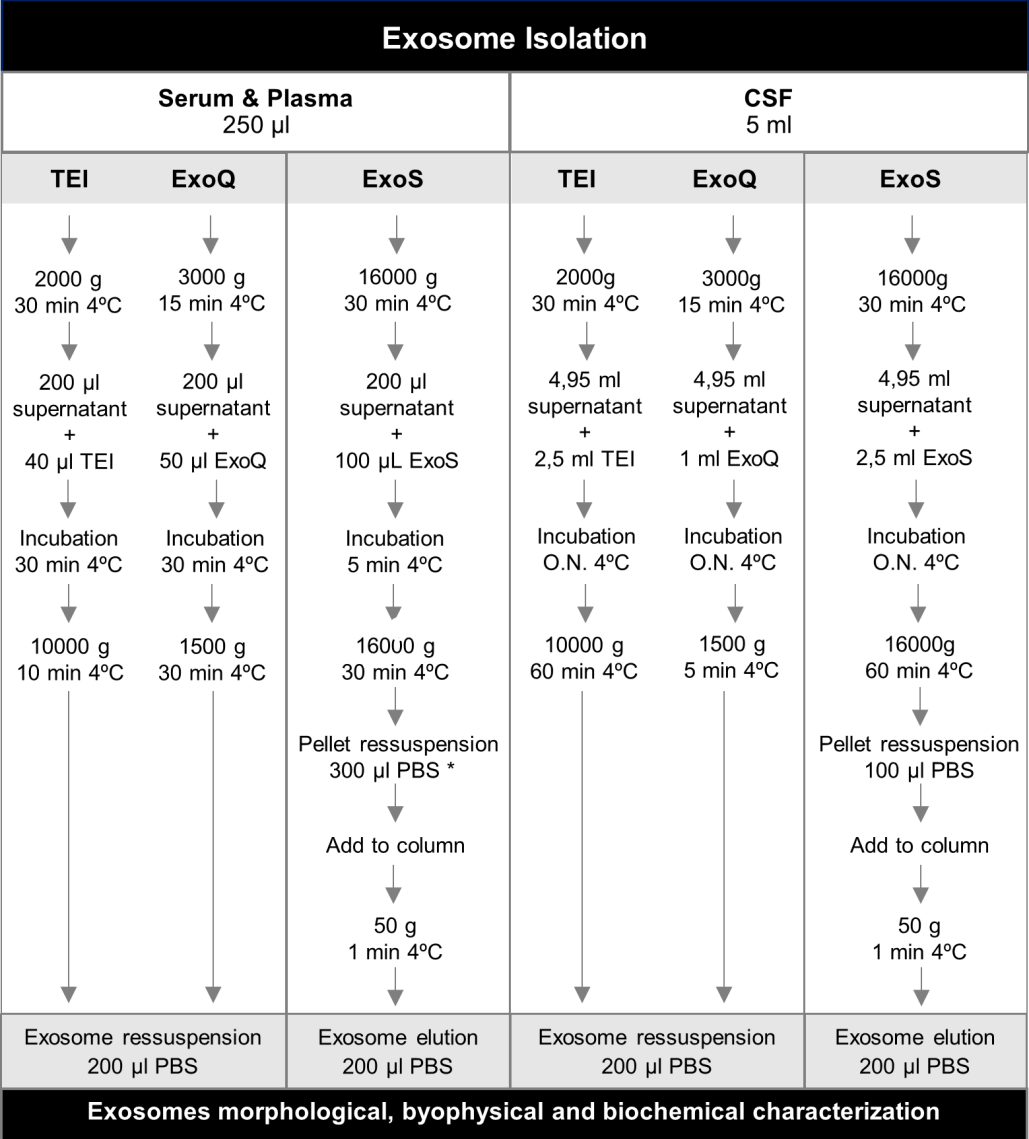
Exosome isolation workflow. Serum and plasma-derived exosomes were isolated using the precipitation-based reagents TEI and ExoQ; and the column-based method ExoS. Pooled exosomes were characterized at different levels and using different methodologies. Exosome extraction from CSF was also performed using equivalent methods. *Serum and plasma-derived exosome preparations obtained using ExoS were at this step ressuspended in higher PBS volumes due to exosome retention in the column.

Exosomes were isolated from a pool of serum and plasma samples using three commercial kits: Total Exosome Isolation™ from serum (Invitrogen) (TEI), ExoQuick™ Serum Exosome Precipitation Solution (System Biosciences) (ExoQ) and Exo-spin™ Blood Exosome Purification Kit (Cell guidance systems) (ExoS). For serum and plasma, exosome purifications were carried out from a 250 μl starting volume (Fig 1). For CSF pool, exosomes were isolated using Total Exosome Isolation™ from cell culture media (Invitrogen) (TEI), ExoQuick-TC™ Exosome Precipitation solution (System Biosciences) (ExoQ) and Exo-spin™ Exosome Purification Kit for cell culture media/urine/saliva and other low-protein biological fluids (Cell guidance systems) (ExoS) (Fig 1). A starting volume of 5 ml of pooled CSF was used for each kit. All samples were prepared according to manufacturer’s instructions with minor modifications, in triplicate for each biofluid and method.

Following exosome isolation and preparation as indicated, exosomes were divided equally into two tubes: one lot of the exosome preparation remained in PBS for exosomal morphology, yield determined through nanoparticle tracking analysis (NTA), stability evaluation (Zetasizer Nano ZS^TM^) and colorimetric particle quantification (EXOCET^TM^); and the other was used for protein quantification (BCA and micro BCA) and immunoblotting (SDS-PAGE and Western Blot). In this case, 100 μl of RIPA buffer (Sigma-Aldrich^TM^) were added to lyse the exosomes; exosome preparations were sonicated 3x, 5 sec/cycle, and prepared for protein quantification and Western Blot analysis as described below. Exosome preparations were stored at -20°C. For transmission electron microscopy (TEM) fresh exosomes were prepared as described.

### Nanoparticle tracking analysis and Electrophoretic Light Scattering

Exosomes’ size distribution curves and concentration measurements were carried out by NTA using a Nanosight NS300^TM^ (Malvern Instruments, UK) equipped with a 488 nm laser and a syringe pump system, with a pump speed of 15. Camera level was set at 11 and an analysis detection threshold of 3. Background measurements were performed with filtered PBS, which revealed the absence of any kind of particles. Three video recordings were carried out for each exosome preparation: for serum and plasma the videos had a duration of 30 sec and for CSF each video had a duration of 40 sec with frame rates of 25 frames/sec. The NTA 3.2 software version was used to record and analyse the sample videos. To evaluate particle stability in solution by Electrophoretic Light Scattering (ELS), Zeta Potential measurements were performed using Zetasizer Nano ZS^TM^ instrument and the software Zetasizer version 7.12 (Malvern Instruments, UK). Zeta potential measurements were carried out at 25°C for each experimental triplicate, biofluid and method. For NTA and zeta potential analysis, exosomes were diluted at 1:1000 in PBS, and sonicated during 10 min in a water-bath at room temperature (RT).

### Transmission electron microscopy

For Transmission Electron Microscopy (TEM), freshly prepared exosomes were isolated as described in Fig 1. Exosomes isolated using TEI and ExoQ were directly fixed in 200 μl of 2% paraformaldehyde. Exosomes isolated using ExoS were eluted in 100 μl of PBS and then fixed with 100 μl of 4% paraformaldehyde (final concentration of 2% paraformaldehyde). In all cases, 20 μl of exosome preparations were allowed to adsorb in a 75 mesh Formvar/carbon coated grid, for 30 min, at RT. Grids were then washed with PBS (membrane side faced down) and dried using a filter paper. For negative staining, exosome-grids were transferred to a 50 μl drop of 3% phosphotungstic acid solution (pH 7) for 10 min and then wicked off with filter paper. TEM visualizations were performed using a Hitachi H-9000 transmission electron microscope at 300 kV and images were captured using a slow-scan CCD camera.

### Colorimetric exosome quantitation

In addition to NTA, exosome concentration was also estimated using the EXOCET^TM^ assay (System Biosciences). This method is based on the measurement of the Acetyl-CoA Acetylcholinesterase enriched activity in exosomes (37). For each preparation condition, 10 μl of exosomal preparation in PBS were diluted in 90 μl of lysis buffer. This dilution was carried out because for serum and plasma, exosomal preparations’ final values were above the standard curve. The mixture was then incubated at 37°C for 5 min, vortexed and centrifuged at 1500 g at RT during 5 min. The subsequent steps of exosome particle quantitation were performed according to manufacturer’s instructions and absorbance was measured at 405 nm using the Infinite M200 plate reader (TECAN^TM^).

### Exosome purity and protein quantification

The purity of exosome preparations was determined by calculating the ratio between particle number, determined by NTA, and protein concentration measured through BCA or Micro BCA assay. Protein quantification of serum and plasma-derived exosome preparations were performed by BCA (Pierce™ BCA Protein Assay kit, Thermo Fisher Scientific), and of CSF-derived exosomes by Micro BCA (Micro BCA™ Protein Assay Kit, Thermo Fisher Scientific). Prior to protein quantification, all exosomes resuspended in PBS were lysed by adding an equal volume of RIPA buffer (Sigma Aldrich) and cOmplete™, Mini, EDTA-free Protease Inhibitor Cocktail^TM^ (Roche), followed by incubation at RT for 5 min and sonicated for 15 seconds. For CSF-derived exosomes, and since RIPA interferes with the Micro BCA assay, exosomes were diluted 1:10 in ultrapure water. In both methods absorbance was read at 562 nm using an Infinite M200 plate reader (TECAN^TM^).

### Western blot analysis

Exosome preparations were lysed by adding an equal volume of RIPA buffer^TM^ (Sigma-Aldrich). Following BCA, preparations were normalized for protein content and 35 μg loaded for each, except for CSF-derived exosomes isolated with ExoQ where half of the concentration was loaded. Loading buffer containing β-mercaptoethanol, was added and exosome preparations boiled at 99°C for 5 min, separated in a 5–20% SDS-polyacrylamide gel gradient (SDS-PAGE) and electrophoretic transferred to nitrocellulose membranes, as previously described [30]. Membranes were blocked in 5% non-fat dry milk in 1x TBS-T (0.5% Tween-20), at RT and incubated with the following primary antibodies: mouse anti-TSG101 (1:500) (612697; BD transduction laboratories^TM^), anti-RAB11 (1:500) (610657; BD transduction laboratories^TM^), anti-NCAM (1:500) (556325; BD transduction laboratories^TM^), anti-albumin (1:500) (sc-374670; Santa Cruz Biotechnology^TM^) or rabbit anti-Calnexin (1:200) (ADI-SPA-860-J). These are commonly used exosome markers. Membranes were then washed with 1x TBS-T (0.5% Tween-20). The secondary antibodies used were the Amersham ECL Mouse IgG, HRP-linked (1:2000) (GE Healthcare Life Sciences^TM^), except in the case of albumin detection where a higher dilution was used (1:5000); and the Amersham ECL Rabbit IgG, HRP-linked (1:5000) (GE Healthcare Life Sciences^TM^). Secondary antibodies were incubated for 2 h, with agitation at RT. Following TBS-T washes, protein bands were detected using the chemiluminescence reagent ECL Select (GE Healthcare Life Sciences^TM^) and images acquired with Chemidoc^TM^ gel imaging system (Bio-Rad).

### Statistical analysis

Statistical analysis was carried out with two-tailed Student’s t-test for methods comparison within each biofluid or Kolmogorov-Smirnov to compare particle size distribution. Only p-values ≤0.05 were considered significant. Error bars in graphs represent standard deviation of the mean for three independent experiments. Analyses were performed using GraphPad Prism 7 (GraphPad Software, La Jolla, California, USA).

## Results

### Exosome size and morphological analysis

The profiles of exosomes derived from different biofluids (serum, plasma and CSF) were evaluated using three commercial kits: two precipitation-based reagents (TEI and ExoQ) and one column-based kit (ExoS). The identification of exosomes focused on various criteria, including size ranging from 30 to 150 nm, confirmation of spherical morphology by TEM and the presence of exosome markers revealed by immunoblotting analysis.

**Fig 2:**
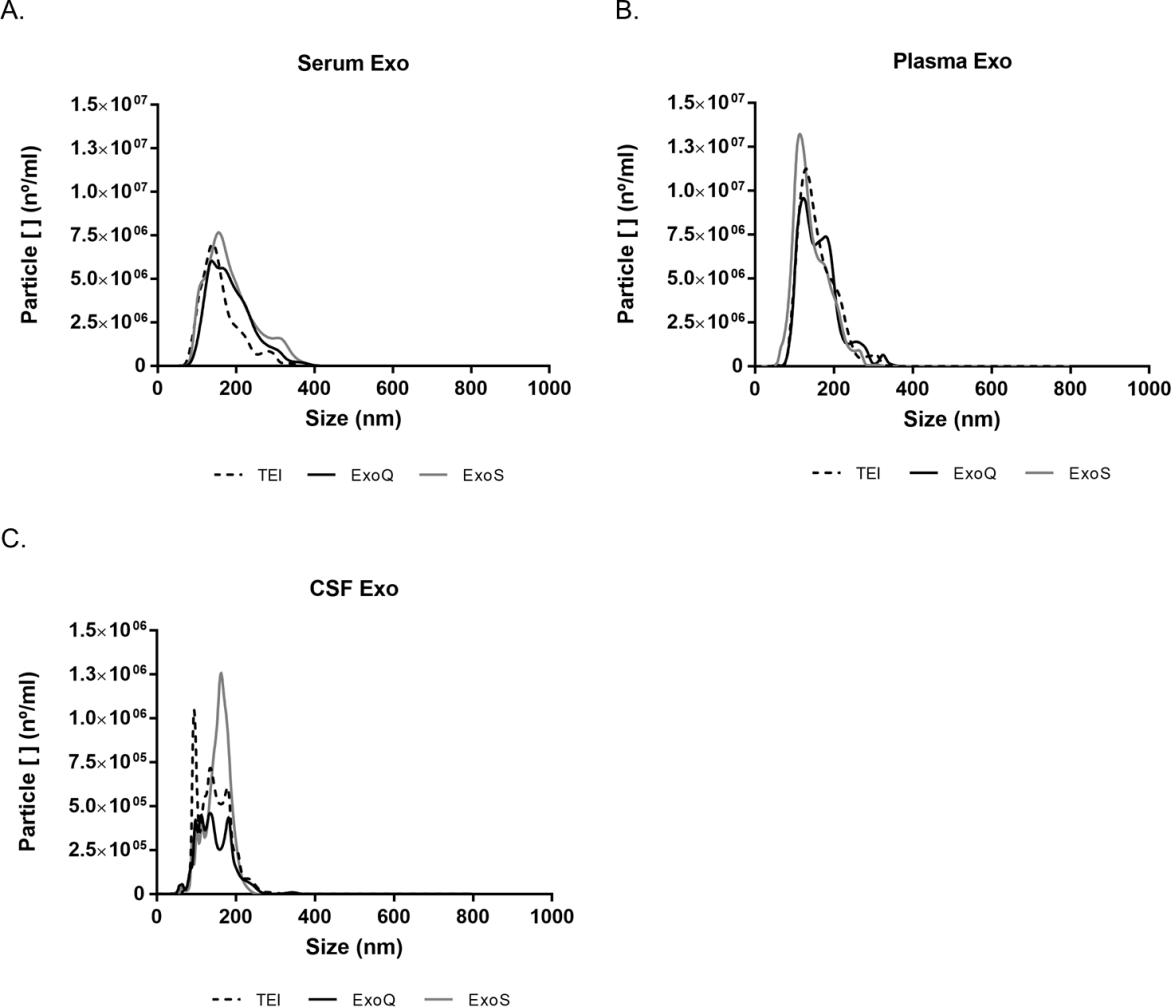
Size profiles of exosomes isolated from distinct biofluids using different methods. Size distribution curves were determined by NTA. (A) Serum, (B) Plasma and (C) CSF. Each curve represents the average of the 3 technical replicate measurements for each exosome isolation method and biofluid triplicate experiment.

Particle size was assessed by NTA for exosomes isolated from the three biofluids using the different kits. Exosomes were also extracted from the distinct biofluids by ultracentrifugation (S1 Fig), but in all cases low exosomal yields were obtained as determined by NTA, consistent with previous reports [31]. Indeed, the number of particles was bellow the NTA detection levels recommended (1x10^7^ particles/ml). For this reason and due to lack of material for comparative purposes in subsequent analysis, the study focused on the precipitation and column-based approaches. All commercial kits resulted in exosomes of identical particle size, with a Gaussian distribution of nanoparticles within the expected size range for exosomes (30–150 nm) (Table 1 and Fig 2). Serum-derived exosomes, isolated with ExoQ, showed a population of EVs with a higher mode tendency than ExoS and TEI (ExoQ 159.3±20.7 nm > ExoS 155.3±24.2 nm > TEI 140.4±20.2 nm) prepared exosomes. In comparative terms, size distributions for curves obtained with TEI and ExoS were similar (p>0.05), and in all cases exosome distribution curves shifted to the right which can be possibly explained by nanoparticles aggregation. Regarding the plasma-derived exosomes, ExoQ also exhibited the highest mode when compared to TEI and ExoS (ExoQ 140.0±28.6 nm > TEI 128.3±9.2 > ExoS 117.6±13.9 nm). Comparatively, exosomes isolated from plasma had different size distribution profiles (p≤0.01) (Table 1 and Fig 2).

**Table 1:** NTA analysis of exosomes pooled.

		Exosome isolation method		
		TEI	ExoQ	ExoS
Serum Exo	Mode (nm)	140,4 ± 20,2	159,3 ± 20,7	155,3 ± 24,2
	Concentration (particles/ml)	5,3x10^8^ ± 1,1x10^8^	5,4x10^8^ ± 0,7x10^8^	6,9x10^8^ ± 0,7x10^8^
Plasma Exo	Mode (nm)	128,3 ± 9,2	140,0 ± 28,6	117,6 ± 13,9
	Concentration (particles/ml)	8,3x10^8^ ± 2,3x10^8^	7,8x10^8^ ± 1,1x10^8^	9,9x10^8^ ± 0,9x10^8^
CSF Exo	Mode (nm)	126,1 ± 40,0	142,6 ± 32,1	155,0 ± 19,8
	Concentration (particles/ml)	6,3x10^7^ ± 1,6x10^7^	4,0x10^7^ ± 0,3x10^7^	6,3x10^7^ ± 1,4x10^7^

Particle concentration and mode values obtained for exosome preparations isolated from serum, plasma and CSF using TEI, ExoQ and ExoS were determined by NTA.

In contrast, CSF nanoparticles isolated with ExoS, presented higher mode value than ExoQ and TEI (ExoS 155.0±19.8 nm > ExoQ 142.6±32.1 nm > TEI 126.1±40.0 nm) (Table 1 and Fig 2). Regarding exosome size distribution profiles TEI and ExoS did not significantly differ (p>0.05), although ExoQ did, suggesting that ExoQ isolated distinct subpopulations of exosomes.

**Fig 3:**
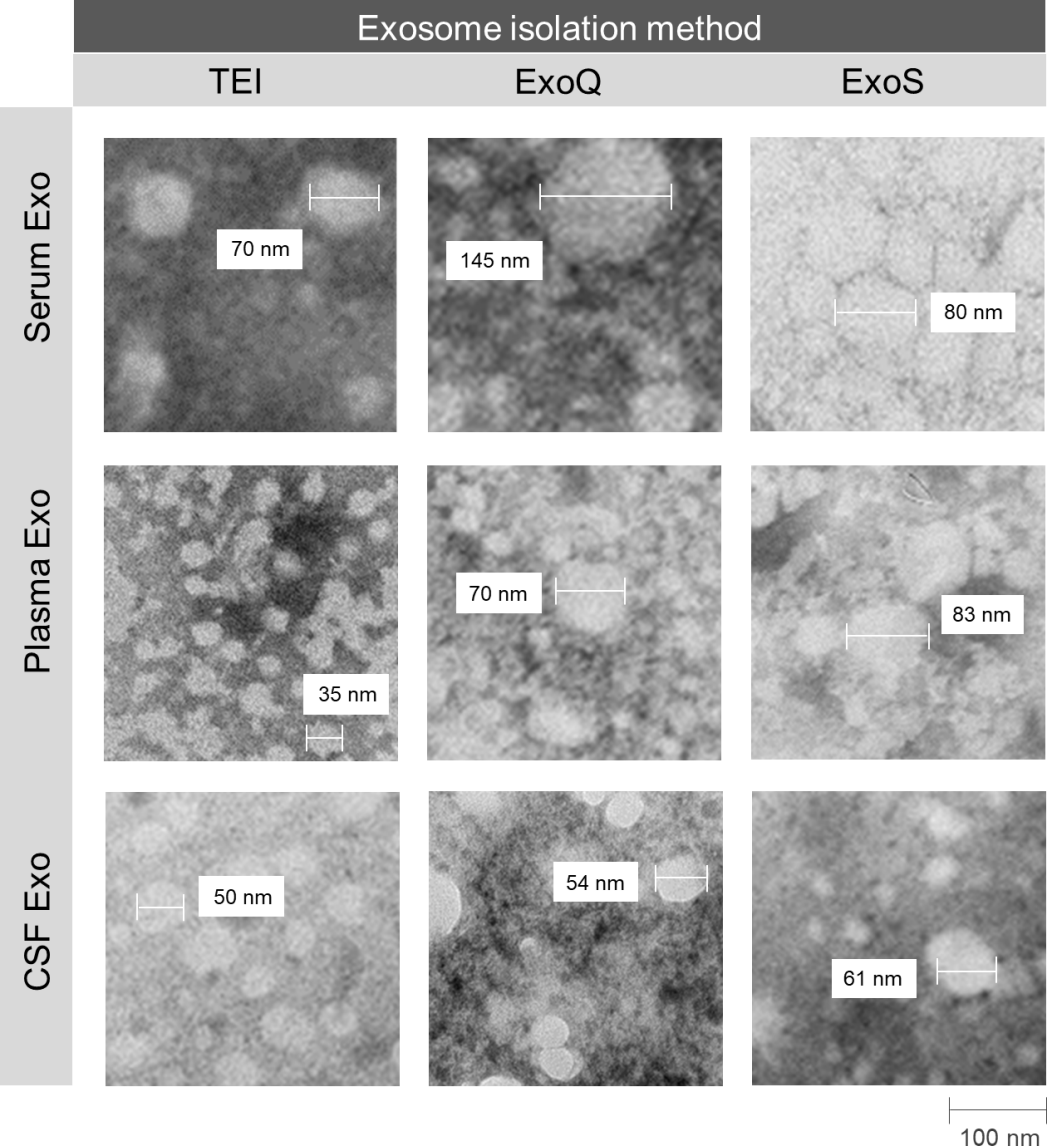
Morphology of exosomes pooled by TEM negative staining. Exosomes were isolated from distinct biofluids using two precipitation-based methods (TEI and ExoQ) and one column-based method (ExoS).

TEM analysis revealed that all techniques successfully isolated exosomes within the expected size range and morphology (Fig 3), consistent with NTA results. In some preparations, microvesicles of smaller size than exosomes and few aggregated like-structures were observed. The latter may explain the increase in size distribution, more frequently found in serum and plasma-derived exosome preparations.

### Exosome yield

**Fig 4:**
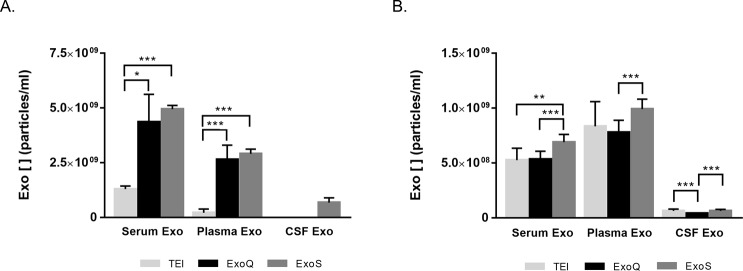
Exosome concentrations determined using different methods. Quantification of exosomes isolated from serum, plasma and CSF by NTA (A) or EXOCET (B). Each bar represents mean values of exosome concentration and error bars indicate standard deviations (n = 9±SD for NTA, obtained from 3 video frames for each biofluid triplicate or n = 6±SD for EXOCET). *p≤0.05, **p≤0.01, ***p≤0.001.

The recovery rate of exosomes generated by the three methods was compared using two different approaches: a biophysical one using NTA and a biochemical one using EXOCET (Fig 4A and 4B, respectively). The latter directly measures acetylcholinesterase enrichment against a standard curve to determine exosome concentration.

For serum derived-exosomes, NTA analysis revealed that ExoS was the most efficient method, isolating 1.3-fold more particles than TEI or ExoQ (p≤0.01). Similarly, in the case of plasma derived exosomes, ExoS also isolated about 1.3-fold more exosomes than ExoQ (p≤0.01). Exosomes isolated from CSF lead to a lower recovery rate of these particles compared with serum and plasma, although higher volumes of starting samples were used (5 ml vs 250 μl). For this biofluid, ExoS and TEI (p≤0.01) render in increased exosome number (Fig 4A and Table 1) comparative to ExoQ.

Contrasting with NTA, EXOCET produces different results in terms of exosome yield. In fact, the number of exosomes obtained with this colorimetric assay was higher than with NTA. Nonetheless, in both techniques a similar pattern for the different isolation methods was observed. For serum and plasma, ExoS was the method yielding higher exosome concentrations, comparative to TEI (p≤0.01), with no differences observed between ExoQ and ExoS performance. This may suggest that exosome vesicle subset isolated with TEI resulted in different biochemical activity than those isolated with ExoS and ExoQ. Consistent with the NTA results, CSF was the biofluid that rendered decreased exosome concentrations. In fact, for TEI and ExoQ the number of exosomes was near the detection limit of this assay, restricting the determination of exosome concentrations. Comparatively, for ExoS, the number of exosomes isolated could be determined although it was very low.

### Exosome zeta potential measurements

**Fig 5:**
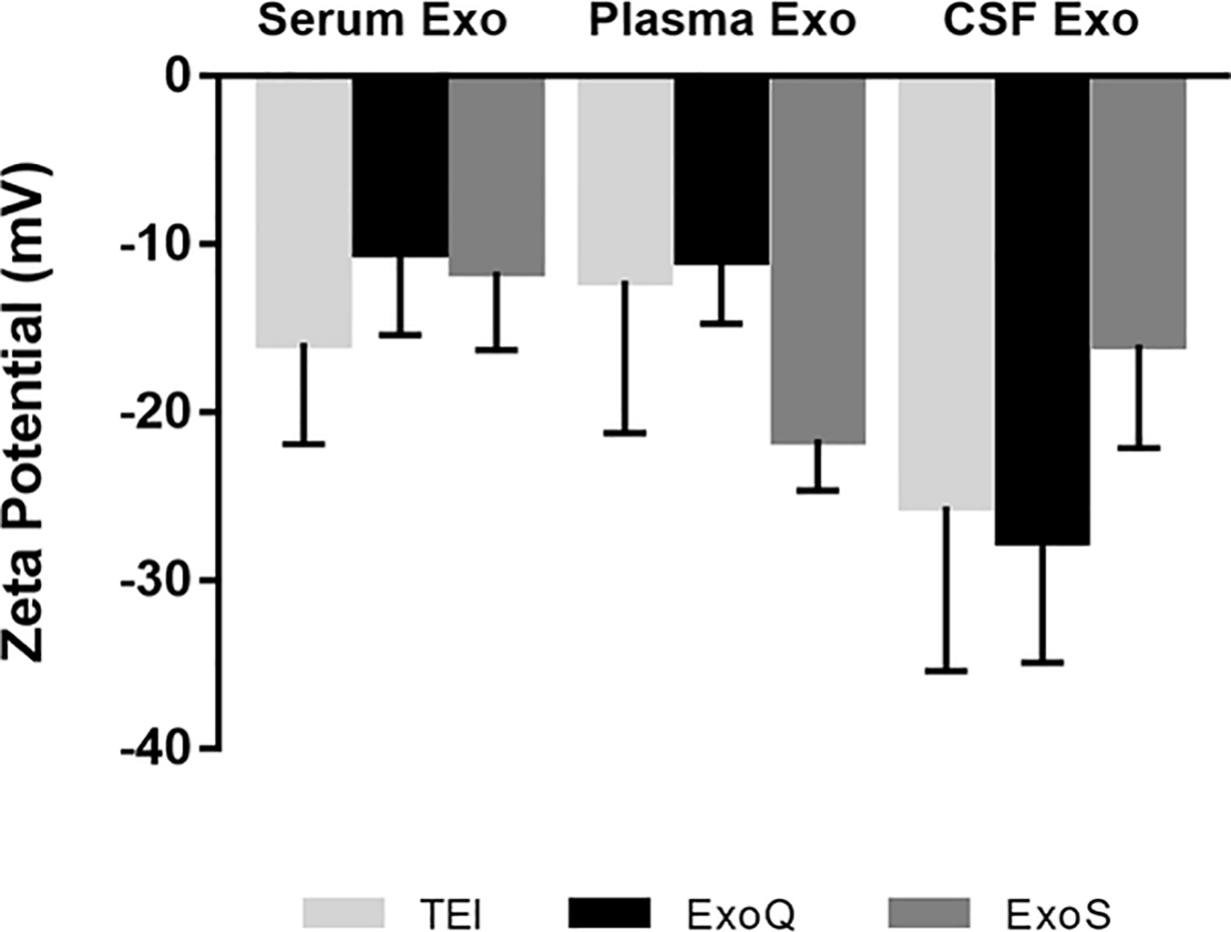
Zeta potential evaluation of exosome stability. Zeta potential was measured in exosomes isolated from serum, plasma and CSF. Each bar represents mean values of zeta potential and error bars indicates standard deviations (n = 3±SD).

To evaluate exosome stability, the zeta potential was measured by ELS. Zeta potentials of all exosome preparations were negative and distributed within the range of -6.3 to -34.3 mV (Fig 5). The conductivity was 16,9 to 17,1 mS/cm, typical for preparations kept in PBS. No significant differences were observed for the three biofluids using the different extraction methods. In general, zeta potential of serum and plasma-derived exosomes was less negative than the one measured for exosomes from the CSF. Despite the low exosome concentration in CSF, and the high conductivity values, this data can be suggestive of greater stability of these exosomes in solution, which might appear to correlate with lower number of particle aggregates.

### Exosome purity and immunoblotting analysis

**Fig 6:**
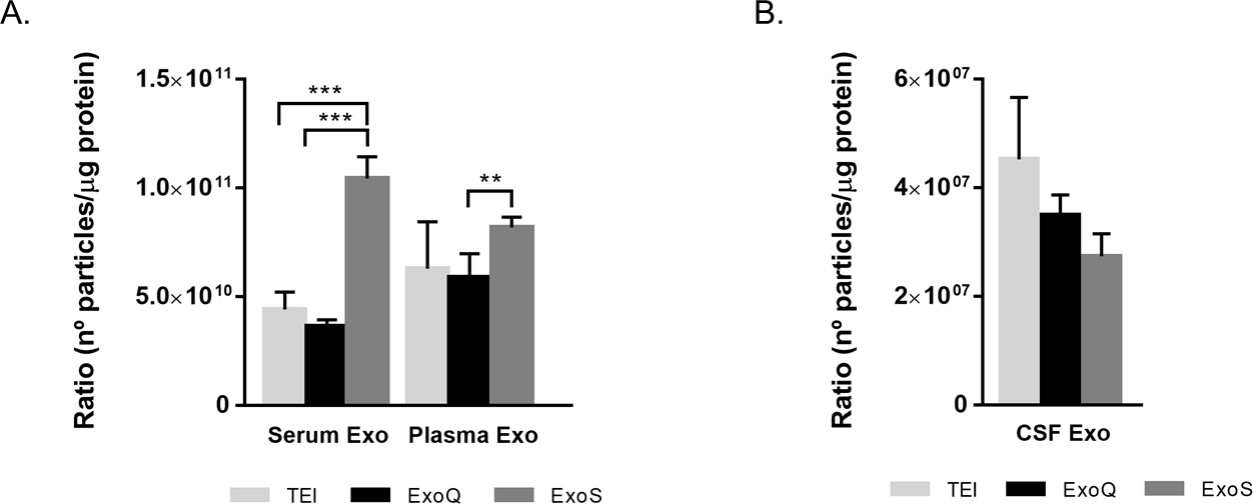
Purity ratio of exosome preparations. Normalization of exosome concentrations determined by NTA per protein concentration measured by BCA for serum- and plasma-derived exosomes isolated and by Micro BCA for CSF-derived exosomes. Each bar represents mean ratio and error bars indicates standard deviations (n = 3±SD). **p≤0.01, ***p≤0.001.

The ratio of particle number to protein concentration has been used to assess exosome purity [32,33]. It has been suggested that highly pure vesicle preparations show particle-to-protein ratios over 3x10^10^ particles per microgram of protein [32]. In this study, protein concentrations in exosome preparations were determined using BCA for serum and plasma-derived exosomes, and using micro BCA for CSF-derived exosomes. Considering this approach, for serum and plasma-derived exosomes, all methods produced highly pure preparations, exceeding purity ratios of 3x10^10^ (Fig 6). Nonetheless, most significant differences in the ratio were found for serum-derived exosomes recovered with ExoS when compared to TEI and ExoQ (p≤0.01). This main difference was due to the higher number of particles and low protein concentrations obtained with ExoS, comparatively to TEI and ExoQ. This suggests that ExoS-derived exosomal preparations apparently have a higher purity. For CSF-derived exosomes, decreased purity ratios were obtained and no significant differences were observed.

**Fig 7:**
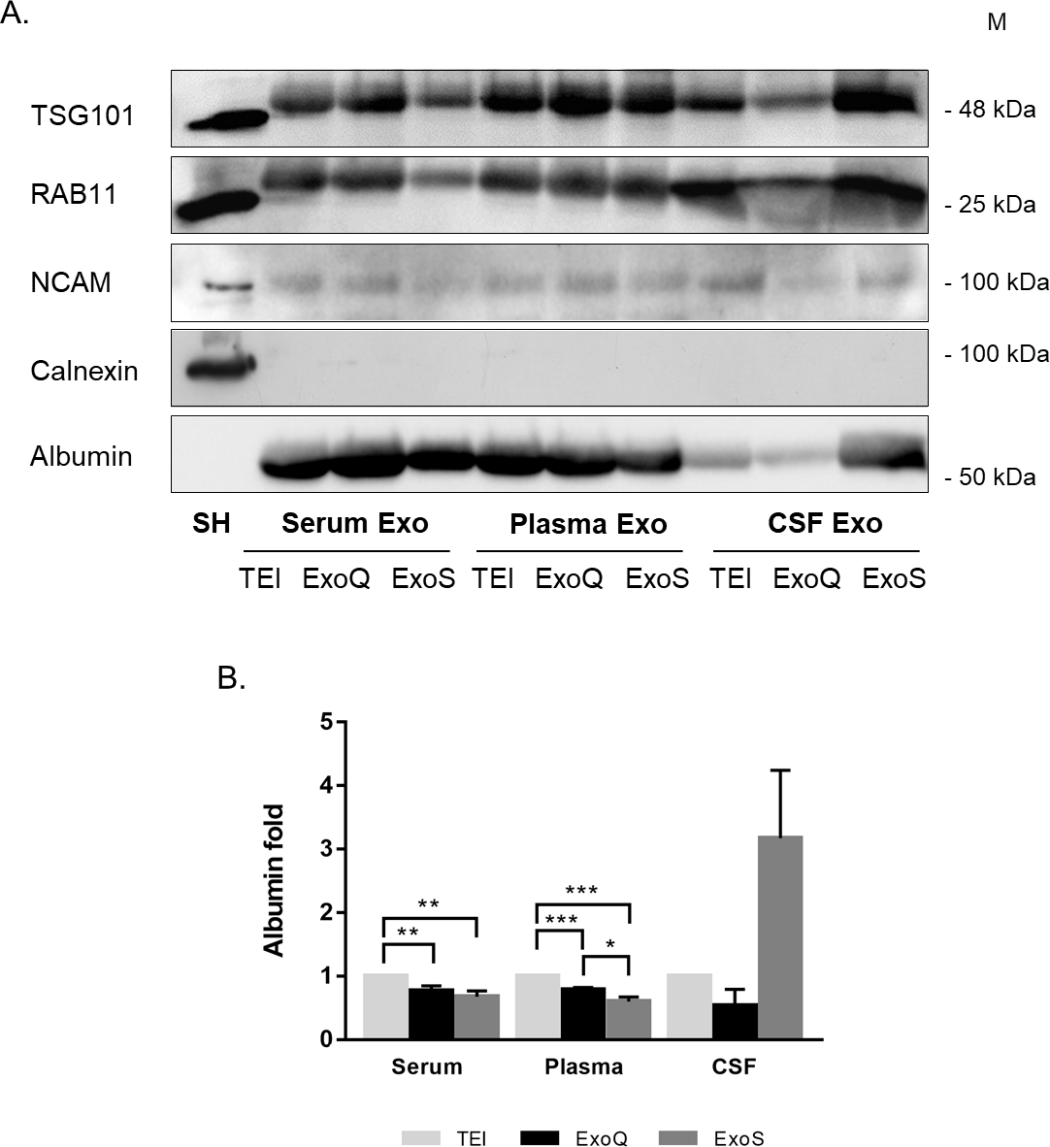
Levels of exosomal markers in pooled exosomes using different methods. **(**A) Western blot analysis for TSG101, RAB11, NCAM, calnexin and albumin in serum, plasma and CSF-derived exosome preparations isolated with the three kits. 35 μg of protein was used from each method, except for CSF ExoQ, were half of the protein concentration was loaded. SH: SH-SY5Y lysates. M: Molecular weight marker. (B) Albumin enrichment in exosomal preparations. Each bar represents mean ratio of three independent experiments and error bars indicates standard deviations (n = 3±SD). *p≤0.05, **p≤0.01, ***p≤0.001.

Complementary analysis of exosomal proteins confirms the presence of exosome markers including TSG101, RAB11 and NCAM, as expected, and the absence of Calnexin (Fig 7A), thus discarding the presence of cell contaminates in the exosomal preparations. However, in all cases some albumin contamination was found (Fig 7B), consistent with previous observations [33,34].

## Discussion

The research interest in exosomes is continuously increasing however the lack of standard methods for isolation and quantification, limits the reliability and reproducibility of exosome use. Different methods have been addressed, with ultracentrifugation being considered the gold standard for exosome isolation. This method is efficient, but laborious and requires higher volumes of human biofluids, not always available and can thus often be a limiting factor. In this study, we compared the performance of three commercial kits, two based on exosome precipitation reagents (TEI and ExoQ) and one column-based method (ExoS), for exosomal isolation from different biofluids namely serum, plasma and CSF. The performance of these kits was addressed by evaluating the number of exosomes isolated, the size, stability and purity. Little is known about these kits performance for exosome isolation, in particular from the CSF.

NTA analysis permitted obtaining size distributions and recovery rates for each exosome preparation, demonstrating that all methods successfully isolated exosomes from serum, plasma and CSF, within the expected range (30–150 nm). It is worthwhile mentioning that NTA technology does not guarantee that particles measured are exclusively exosomes, just that these particles are within the expected size range. NTA measures the individual hydrodynamic diameter based in Stokes Einstein equation, which is influenced by size, thus this technique is also sensitive to the presence of higher particles or aggregates.

In our study, size distribution curves per concentration of particles were distinct for the three methods and biofluids, indicating that exosome preparations exhibit particles of distinct sizes and that kits have different isolation performances, which can render in different exosome subpopulations [35]. Most particles were in the expected size range; but size distribution curves showed a percentage of particles (around 10% for serum and plasma and 25% for CSF) superior to 200 nm. This might relate to the presence of some of non-exosomal vesicles [36] or alternatively to the presence of exosome aggregates. Consistently, for some TEM preparations, aggregated exosome-like structures were detected which can explain the peaks of larger diameter obtained by NTA. Previous studies reported similar results using precipitation-based reagents for exosome isolation from serum and plasma and NTA, or different methodologies such as dynamic light scattering [31,34,37,38]. Efforts have been made to overcome the exosome aggregation effect, and indeed for cell culture media-derived exosomes trehalose and PBS was used to reduce exosome aggregation [38], however, to our knowledge, this had never been previously tested in biofluids. Furthermore, a population of particles, below 30 nm, was evident and observed in TEM preparations representing around 1% of NTA particles. These may reflect contamination with high-density, low-density and/or intermediate-density lipoproteins, as previously suggested [39]. Nonetheless, TEM analysis also revealed nanovesicle typical sizes and morphology for all exosome preparations; and immunoblotting analysis confirmed the presence of exosomes by the detection of TSG101 and RAB11 exosomal markers.

Further analysis included exosome quantification using NTA and EXOCET assay, the latter is a method based on the characteristically enhanced activity of exosome acetyl-CoA acetylcholinesterase, enriched in exosomes [40]. Exosome recovery rates determined by NTA were significantly different between the three kits, with ExoS performing better (p≤0.01) for serum and plasma than the other methods. The number of exosomes isolated from serum and plasma was quite similar to concentrations previously reported, using NTA [31,34,41,42]. For CSF, and although the initial starting volume was increased, the recovery rates were very low. CSF is a biofluid that usually renders in decrease exosomal yields, even when using different isolation methods [6,43].

Comparatively to NTA, the EXOCET method renders in increased exosome concentrations, in particular for ExoS and ExoQ, consistent with a previously report on exosomes isolated from human serum [44]. This is probably related to the different nature of the methods (biophysical versus biochemical/enzymatic) and/or to the different extracellular vesicle subsets isolated by the distinct procedures. TEI resulted in a decrease in exosome recovery rates using EXOCET, what might be explained by differences in the biochemical activity of exosomes isolated by this approach. Nonetheless, in general, this quantification method highlighted the best performance of the ExoS for isolating exosomes, particularly from serum and plasma. Integrating all the quantification methods, ExoS rendered in the most efficient in isolating exosomes from the different biofluids.

Additionally, zeta potential analysis was carried out to address exosome preparation stability, and although variations in zeta potential were observed within biofluid and isolation methods, no significant differences were identified. Exosome preparations were negatively charged and within the range previously reported [31,45,46], with most values obtained bellow -10 mV (the minimum threshold value commonly accepted for sample stability in dispersion). Samples with smaller zeta potential than this are highly prone to aggregation. Particles with higher charges are less likely to aggregate and are much more stable in dispersion [47]; a tendency observed mostly for CSF. Exosome preparations exhibiting lower concentrations will be less prone to aggregate, and thus potentially more stable. There are still limitations when interpreting the results of exosome zeta potentials, however it is reported that exosome storage period and temperature impact exosome stability, recovery and can even lead to exosome markers loss as well as morphological changes [48].

The purity of exosomal preparations was determined as a ratio of particle number to protein concentration, as previously reported [32,33]. In general, for serum and plasma all kits isolated exosomes with similar purity, although ExoS provided the highest purity, consistent with previous results for column-derived exosomal preparations [33]. Nonetheless, exosome preparations are not entirely pure but rather enriched in exosomes, as expected. The presence of albumin suggested exosomal sample contamination, in accordance with previous reports [33,34]. Although, blood-derived exosomes isolated with ExoS have tendentiously less albumin contamination and thus consistent with highly pure preparations, exosome markers were also less enriched. This can be explained by the difference in extracellular vesicles subsets and corresponding protein cargo (including exosomal markers) recovered by each method [35].

In conclusion, our study showed that both precipitation-based reagents and the column-based kit isolated exosomes from the distinct biofluids (Table 2). However, these kits performed differently in terms of size distributions, number of exosomes obtained and level of purity. In general, the higher performance and purity was obtained with ExoS for serum and plasma-derived exosomes. For CSF, this kit also produced a higher exosomal recovery. In general, the methods herein described represent quicker and reliable alternatives for exosome isolation from reduced biofluids volumes, particularly from serum or plasma, validating the use of these kits in clinical research. As exosomes can act as a contained resource for biomarkers, the choice of the most adequate exosome isolation methods and biofluids will contribute not only to the expansion of the biomarker discovery field but can also impact on the expansion of drug delivery systems for a wide range of diseases.

**Table 2:** Qualitative comparison of the kits performance in exosome isolation from distinct biofluids.

	Serum			Plasma			CSF		
	TEI	ExoQ	ExoS	TEI	ExoQ	ExoS	TEI	ExoQ	ExoS
Exosome yield (NTA)	**	**	***	**	**	***	*	*	*
Exosome yield (EXOCET)	*	***	***	*	**	**	-	-	*
Ratio (n° particles/μg protein)	**	**	***	**	**	***	*	*	*

*Low yield or purity

** Medium yield or purity

*** High yield or purity;—No yield.

## Supporting information

S1 Fig Overall characterization of ultracentrifugation (UC) performance in exosome isolation from distinct biofluids. A starting volume of 250 μl of serum and plasma was centrifuged at 2000 g, 30 min, at 4°C. The supernatant was then diluted in PBS and ultracentrifuged using a Beckman Coulter Optima XE-100 Ultracentrifuge at 110000 g, 2h, 4°C followed by pellet ressuspension and centrifugation at 110000 g, 1h, 4°C. For CSF, 5 ml of biofluid was used and prepared as above, except for the first steps of UC that was carried out at 110000 g only for 1 h, at 4°C. For all biofluids the final volume of resuspension was 200 μl of PBS. (A) Size profiles of exosomes isolated. Size curves were determined by nanoparticle tracking analysis using a Nanosight NS300TM. Three video recordings of 40 sec were carried out for each preparation, diluted at 1:1000 in PBS. NTA 3.2 software version was used to record and analyse the videos. (B) TEM morphology of exosomes isolated. TEM visualizations were performed using a Hitachi STEM HD2700 at 200 kV and images captured with a slow-scan CCD camera. (C) Quantification of exosomes isolated using NTA. Each bar represents mean values of exosome concentration and error bars indicates standard deviations (n = 9±SD, 3 technical replicates for the 3 exosomal preparations per each method and biofluid). (D) Purity ratio of exosome preparations. Normalization of serum, plasma and CSF exosome concentrations determined by NTA per protein concentration measured by Micro BCA assay. Each bar represents mean ratio and error bars indicates standard deviations (n = 3±SD). (E) Western blot analysis of exosomes preparations for TSG101, RAB11, NCAM, calnexin and albumin. 20 μg of protein was loaded for each preparation and resolved in a 5–20% SDS-PAGE gel, electrophoretic transferred and immunoblotted for exosomal markers. SH:SH-SY5Y lysates. M: Molecular weight marker. S:Serum P:Plasma CSF:Cerebrospinal fluid.
(TIF)
