# Supplementary figures and images for: Exosome isolation from distinct biofluids using precipitation and column-based approaches
Source: PLoS One. 2018 Jun 11;13(6):e0198820. doi: 10.1371/journal.pone.0198820 (PMC5995457; doi:10.1371/journal.pone.0198820)

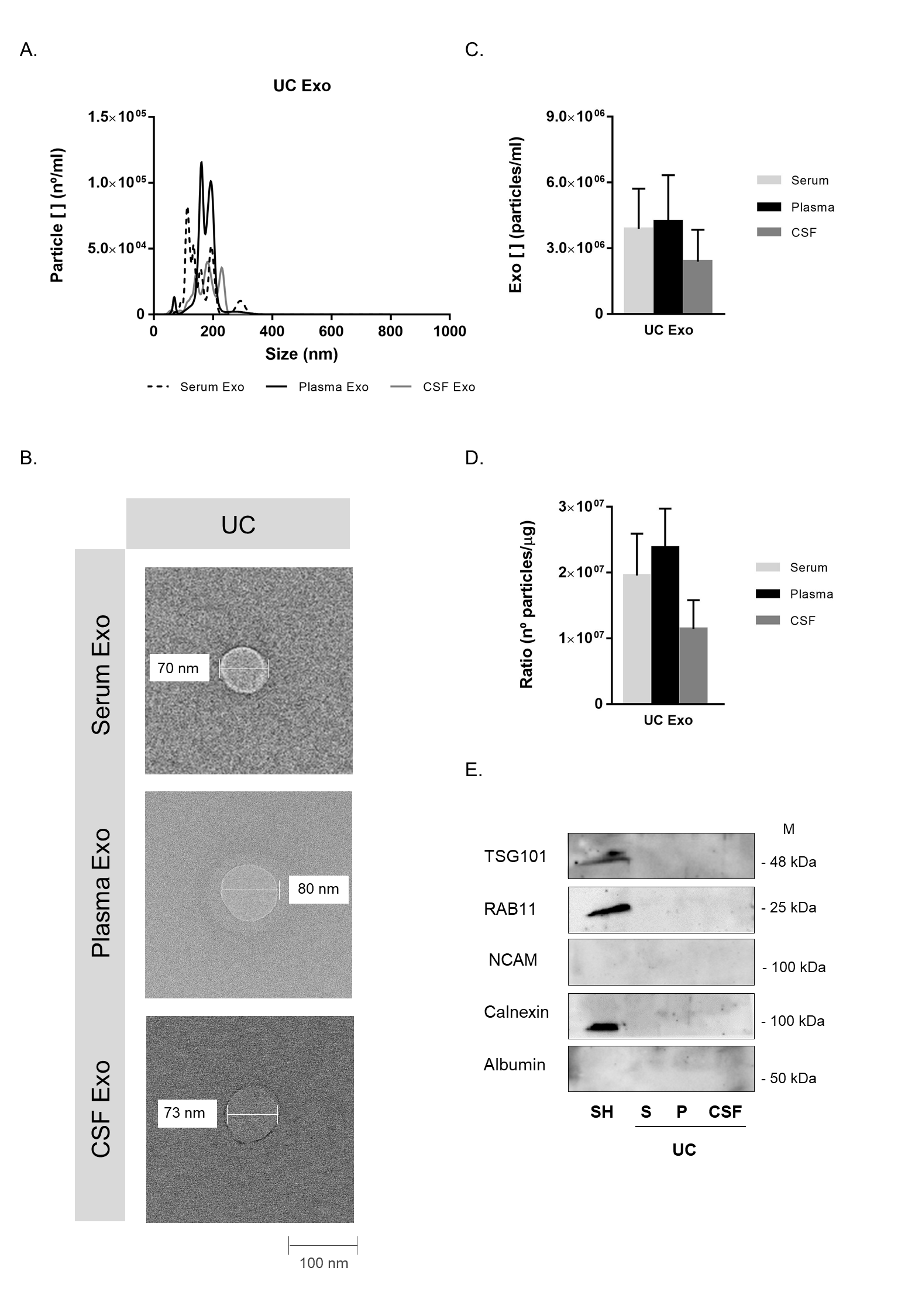

Supplement: S1 Fig — A starting volume of 250 μl of serum and plasma was centrifuged at 2000 g, 30 min, at 4°C. The supernatant was then diluted in PBS and ultracentrifuged using a Beckman Coulter Optima XE-100 Ultracentrifuge at 110000 g, 2h, 4°C followed by pellet ressuspension and centrifugation at 110000 g, 1h, 4°C. For CSF, 5 ml of biofluid was used and prepared as above, except for the first steps of UC that was carried out at 110000 g only for 1 h, at 4°C. For all biofluids the final volume of resuspension was 200 μl of PBS. (A) Size profiles of exosomes isolated. Size curves were determined by nanoparticle tracking analysis using a Nanosight NS300TM. Three video recordings of 40 sec were carried out for each preparation, diluted at 1:1000 in PBS. NTA 3.2 software version was used to record and analyse the videos. (B) TEM morphology of exosomes isolated. TEM visualizations were performed using a Hitachi STEM HD2700 at 200 kV and images captured with a slow-scan CCD camera. (C) Quantification of exosomes isolated using NTA. Each bar represents mean values of exosome concentration and error bars indicates standard deviations (n = 9±SD, 3 technical replicates for the 3 exosomal preparations per each method and biofluid). (D) Purity ratio of exosome preparations. Normalization of serum, plasma and CSF exosome concentrations determined by NTA per protein concentration measured by Micro BCA assay. Each bar represents mean ratio and error bars indicates standard deviations (n = 3±SD). (E) Western blot analysis of exosomes preparations for TSG101, RAB11, NCAM, calnexin and albumin. 20 μg of protein was loaded for each preparation and resolved in a 5–20% SDS-PAGE gel, electrophoretic transferred and immunoblotted for exosomal markers. SH:SH-SY5Y lysates. M: Molecular weight marker. S:Serum P:Plasma CSF:Cerebrospinal fluid. (TIF) [file pone.0198820.s001.tif]
